# Supplementary material for: Proxies for use in biochar decay models: Hydropyrolysis, electric conductivity, and H/Corg molar ratio
Source: PLoS One. 2025 Sep 2;20(9):e0330206. doi: 10.1371/journal.pone.0330206 (PMC12404433; doi:10.1371/journal.pone.0330206)
Supplement: S1 Table — Analytical methods used and data on the composition of the biomass used for biochar production. LOQ = limit of quantification. (PDF) [file pone.0330206.s001.pdf]

S1 Table of feedstock composition. Analytical methods used and data on the composition of the biomass used for biochar production.

LOQ = limit of quantification.

	Method	LOQ		Softwood	Straw
Ash (550°C)	DIN EN ISO 18122: 2016-03	0.1	Mass%	0.4	5.9
Carbon (C)	DIN EN ISO 16948: 2015-09	0.2	Mass%	50.3	43.8
	2015-09				
Main and trace elements (DIN EN ISO 16967:2015-07 bzw. DIN EN ISO 16968:2015-09)					
Arsenic (As)	DIN EN ISO 17294-2 (E29): 2017-01	0.8	mg/kg	< 0.8	< 0.8
Lead (Pb)	DIN EN ISO 17294-2 (E29): 2017-01	2	mg/kg	< 2	< 2
Boron (B)	DIN EN ISO 17294-2 (E29): 2017-01	2	mg/kg	2	3
Cadmium (Cd)	DIN EN ISO 17294-2 (E29): 2017-01	0.2	mg/kg	< 0.2	< 0.2
Chromium (Cr)	DIN EN ISO 17294-2 (E29): 2017-01	1	mg/kg	< 1	6
Copper (Cu)	DIN EN ISO 17294-2 (E29): 2017-01	1	mg/kg	< 1	2
Manganese (Mn)	DIN EN ISO 17294-2 (E29): 2017-01	1	mg/kg	104	18
Nickel (Ni)	DIN EN ISO 17294-2 (E29): 2017-01	1	mg/kg	< 1	2
Mercury (Hg)	DIN EN ISO 12846 (E12):2012-08	0.05	mg/kg	< 0.05	< 0.05
Zinc (Zn)	DIN EN ISO 17294-2 (E29): 2017-01	1	mg/kg	4	9
Elements from the digestion of the ash (550°C) according to DIN 51729-11:1998-11					
Aluminium as Al_2O_3	DIN EN ISO 11885 (E22): 2009-09	0.1	Mass% of ash	0.6	0.6
Calcium as Calciumoxid	DIN EN ISO 11885 (E22): 2009-09	0.1	Mass% of ash	33.2	12.2
Iron as Fe_2O_3	DIN EN ISO 11885 (E22): 2009-09	0.1	Mass% of ash	1	0.5
Potassium as K_2O	DIN EN ISO 11885 (E22): 2009-09	0.1	Mass% of ash	12.3	32.6
Magnesium as MgO	DIN EN ISO 11885 (E22): 2009-09	0.1	Mass% of ash	5.5	0.8
Manganese as MnO	DIN EN ISO 11885 (E22): 2009-09	0.1	Mass% of ash	3.2	< 0.1
Sodium as Na_2O	DIN EN ISO 11885 (E22): 2009-09	0.1	Mass% of ash	1	0.5
Phosphorous as P_2O_5	DIN EN ISO 11885 (E22): 2009-09	0.1	Mass% of ash	2.5	1.4
Sulfur as SO_3	DIN EN ISO 11885 (E22): 2009-09	0.1	Mass% of ash	2.7	23.4
Silicon as SiO_2	DIN EN ISO 11885 (E22): 2009-09	0.1	Mass% of ash	2.7	15.2
Titanium as TiO_2	DIN EN ISO 11885 (E22): 2009-09	0.1	Mass% of ash	0.1	< 0.1
