# Supplementary material for: Proxies for use in biochar decay models: Hydropyrolysis, electric conductivity, and H/Corg molar ratio
Source: PLoS One. 2025 Sep 2;20(9):e0330206. doi: 10.1371/journal.pone.0330206 (PMC12404433; doi:10.1371/journal.pone.0330206)
Supplement: S1 Fig — Wood pellets used for pyrolysis. Pens serve as a size reference. Pellets for the production of biochars at 400–600 °C (right) were more dense than the other pellets (left). (PDF) [file pone.0330206.s002.pdf]

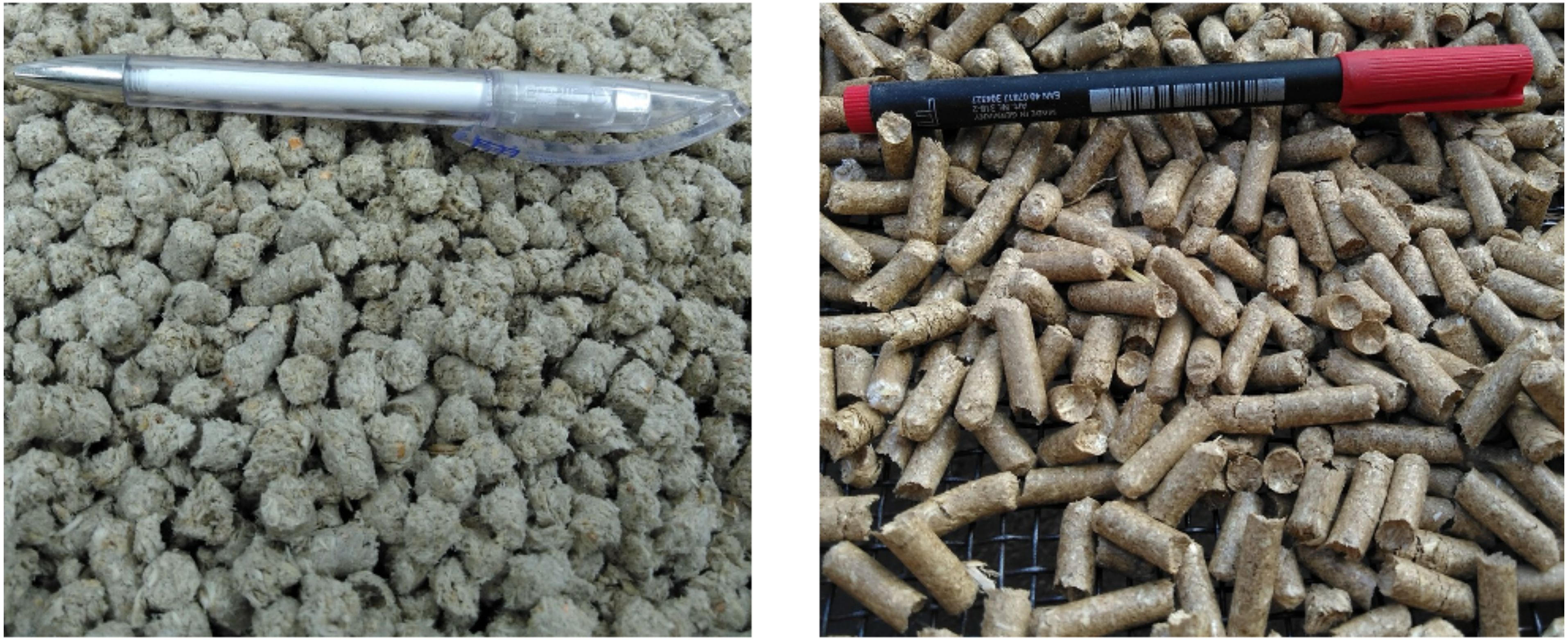

S2 Figure of feedstock pellets. Wood pellets used for pyrolysis. Pens serve as a size reference. Pellets for the production of biochars at 400-600°C (right) were more dense than the other pellets (left).
