# Supplementary material for: Proxies for use in biochar decay models: Hydropyrolysis, electric conductivity, and H/Corg molar ratio
Source: PLoS One. 2025 Sep 2;20(9):e0330206. doi: 10.1371/journal.pone.0330206 (PMC12404433; doi:10.1371/journal.pone.0330206)
Supplement: S2 Table — Content of total carbon (TC), hydrogen (H), H/C molar ratio, BCHyPy as part of total carbon (TC), BCHyPy of total biochar mass, BCHyPy of the dry and ash free (daf) of biochar, and solid-state electric conductivity (SEC). Biochars were produced from wood (W) and straw (S) pellets at 400–800 °C as indicated in the sample name. (PDF) [file pone.0330206.s003.pdf]

S3 Table: Properties of biochar. Content of total carbon (TC), hydrogen (H), H/C molar ratio, BC_{HyPy} as part of total carbon (TC), BC_{HyPy} of total biochar mass, BC_{HyPy} of the dry and ash free (daf) of biochar, and solid-state electric conductivity (SEC). Biochars were produced from wood (W) and straw (S) pellets at 400-800 °C as indicated in the sample name.

	TC (%)	H (%)	H/C Molar ratio	Ash (%)	BC_{HyPy} (% of TC)	BC_{HyPy} (Mass%)	Conductivity (mS cm^{-1}) at 30 MPa	Conductivity (mS cm^{-1}) at 60 MPa	Conductivity (mS cm^{-1}) at 90 MPa	Conductivity (mS cm^{-1}) at 125 MPa	Conductivity (mS cm^{-1}) at 160 MPa
S400[1]	62.1	2.5	0.48	27.5	46.7	29.0	4.5 x 10^{-5}	5.5 x 10^{-5}	6.5 x 10^{-5}	7.9 x 10^{-5}	7.6 x 10^{-5}
S450[1]	67.9	2.4	0.42	22.3	65.9	44.7	3.7 x 10^{-4}	4.8 x 10^{-4}	5.6 x 10^{-4}	6.5 x 10^{-4}	6.8 x 10^{-4}
S500[1]	69.0	2.5	0.44	21.8	77.1	53.2	3.4 x 10^{-3}	4.3 x 10^{-3}	5.0 x 10^{-3}	5.7 x 10^{-3}	6.1 x 10^{-3}
S550[1]	69.7	2.0	0.34	22.2	78.2	54.5	5.2 x 10^{-2}	6.6 x 10^{-2}	8.3 x 10^{-2}	9.6 x 10^{-2}	1.0 x 10^{-1}
S600[1]	69.3	1.7	0.30	22.6	85.6	59.3	1.7 x 10^{-1}	2.2 x 10^{-1}	2.5 x 10^{-1}	2.8 x 10^{-1}	2.9 x 10^{-1}
S620[2]	69.4	1.5	0.25	22.9	87.0	60.4	7.8 x 10^{-1}	9.7 x 10^{-1}	1.1 x 10^{0}	1.2 x 10^{0}	1.3 x 10^{0}
S640[2]	68.5	1.4	0.25	22.8	84.3	57.8	2.3 x 10^{0}	3.1 x 10^{0}	3.6 x 10^{0}	3.9 x 10^{0}	4.1 x 10^{0}
S660[2]	68.3	1.3	0.22	23.7	89.2	60.9	1.3 x 10^{1}	1.7 x 10^{1}	1.9 x 10^{1}	2.2 x 10^{1}	2.3 x 10^{1}
S680[2]	68.4	1.2	0.20	24.1	92.0	62.9	3.9 x 10^{1}	5.2 x 10^{1}	5.7 x 10^{1}	6.4 x 10^{1}	6.9 x 10^{1}
S700[2]	68.1	1.1	0.20	24.4	90.6	61.7	6.0 x 10^{1}	8.0 x 10^{1}	8.8 x 10^{1}	9.9 x 10^{1}	1.1 x 10^{2}
S720[2]	67.8	0.9	0.17	24.9	95.9	65.0	1.7 x 10^{2}	2.2 x 10^{2}	2.4 x 10^{2}	2.7 x 10^{2}	2.9 x 10^{2}
S740[2]	67.4	1.0	0.17	25.2	97.0	65.3	3.0 x 10^{2}	4.0 x 10^{2}	4.4 x 10^{2}	4.9 x 10^{2}	5.3 x 10^{2}
S760[2]	67.0	0.9	0.16	25.2	95.1	63.7	4.7 x 10^{2}	6.3 x 10^{2}	7.0 x 10^{2}	7.6 x 10^{2}	8.2 x 10^{2}
S780[2]	61.9	1.3	0.25	26.5	90.3	55.9	6.5 x 10^{2}	8.2 x 10^{2}	9.1 x 10^{2}	1.0 x 10^{3}	1.1 x 10^{3}
S800[2]	64.4	0.8	0.16	25.3	92.5	59.6	8.4 x 10^{2}	1.1 x 10^{3}	1.2 x 10^{3}	1.3 x 10^{3}	1.4 x 10^{3}
W400[1]	81.2	2.9	0.43	1.7	59.0	47.9	1.6 x 10^{-5}	2.0 x 10^{-5}	2.5 x 10^{-5}	2.8 x 10^{-5}	3.0 x 10^{-5}
W450[1]	83.8	2.9	0.41	1.5	59.8	50.1	5.8 x 10^{-5}	7.7 x 10^{-5}	8.9 x 10^{-5}	1.0 x 10^{-4}	1.1 x 10^{-4}
W500[1]	86.7	3.0	0.42	2.1	78.8	68.3	2.0 x 10^{-4}	2.8 x 10^{-4}	3.4 x 10^{-4}	4.0 x 10^{-4}	4.5 x 10^{-4}
W550[1]	88.6	2.5	0.34	2.0	77.2	68.4	1.0 x 10^{-3}	1.3 x 10^{-3}	1.5 x 10^{-3}	1.7 x 10^{-3}	1.8 x 10^{-3}
W600[1]	88.9	2.4	0.32	2.1	91.2	81.1	2.3 x 10^{-3}	3.2 x 10^{-3}	4.0 x 10^{-3}	4.8 x 10^{-3}	5.5 x 10^{-3}
W620[2]	88.9	2.2	0.29	3.1	88.2	78.3	1.3 x 10^{-2}	1.8 x 10^{-2}	2.2 x 10^{-2}	2.7 x 10^{-2}	2.9 x 10^{-2}
W640[2]	88.9	2.0	0.27	3.3	91.9	81.7	9.8 x 10^{-2}	1.3 x 10^{-1}	1.6 x 10^{-1}	1.9 x 10^{-1}	2.1 x 10^{-1}
W660[2]	88.0	2.0	0.27	3.3	93.1	81.9	7.2 x 10^{-1}	9.8 x 10^{-1}	1.1 x 10^{-1}	1.3 x 10^{-1}	1.5 x 10^{-1}

W680[2]	88.5	1.7	0.23	3.0	89.6	79.3	5.0×10^{0}	7.1×10^{0}	8.2×10^{0}	9.5×10^{0}	1.0×10^{0}
W700[2]	89.8	1.5	0.21	5.0	92.2	82.8	1.9×10^{1}	2.6×10^{1}	3.4×10^{1}	3.6×10^{1}	4.3×10^{1}
W720[2]	90.4	1.3	0.17	2.9	97.4	88.0	3.4×10^{1}	4.7×10^{1}	5.8×10^{1}	6.5×10^{1}	7.7×10^{1}
W740[2]	91.0	1.2	0.16	2.8	93.4	84.9	1.8×10^{2}	2.5×10^{2}	2.9×10^{2}	3.5×10^{2}	3.6×10^{2}
W760[2]	90.8	1.2	0.16	2.6	95.4	86.6	4.4×10^{2}	6.0×10^{2}	7.3×10^{2}	8.3×10^{2}	9.6×10^{2}
W780[2]	91.5	0.9	0.12	2.7	98.5	90.1	8.4×10^{2}	1.2×10^{3}	1.6×10^{3}	1.7×10^{3}	2.0×10^{3}
W800[2]	91.8	0.8	0.10	2.8	91.3	83.8	1.5×10^{3}	1.9×10^{3}	2.5×10^{3}	2.7×10^{3}	3.2×10^{3}

[1]*: biochar produced with the first batches of pellets.*

[2]*: biochar produced with the second batches of pellets.*
