# Supplementary material for: Proxies for use in biochar decay models: Hydropyrolysis, electric conductivity, and H/Corg molar ratio
Source: PLoS One. 2025 Sep 2;20(9):e0330206. doi: 10.1371/journal.pone.0330206 (PMC12404433; doi:10.1371/journal.pone.0330206)
Supplement: Attachment — Submitted filename: PONE-D-25-30085_reviewer.pdf [file pone.0330206.s004.pdf]

PLOS ONE

Proxies for use in Biochar Decay Models: Hydropyrolysis, Electric Conductivity, and H/Corg molar ratio

--Manuscript Draft--

**Manuscript Number:**	PONE-D-25-30085
**Article Type:**	Research Article
**Full Title:**	Proxies for use in Biochar Decay Models: Hydropyrolysis, Electric Conductivity, and H/Corg molar ratio
**Short Title:**	Hydropyrolysis, Electric Conductivity, and H/Corg molar ratio of biochar
**Corresponding Author:**	Nikolas Hagemann<br>Agroscope Standort Reckenholz<br>Tuebingen, SWITZERLAND
**Keywords:**	pyrogenic carbon; persistence; carbon dioxide removal; PyCCS,
**Abstract:**	Biochar is a carbon-rich material produced via pyrolysis that is increasingly recognized for its role in carbon sequestration, particularly through its application in agriculture and materials. However, accurately predicting the long-term persistence of biochar in the environment remains challenging. While incubation trials have been widely used to assess biochar degradation, their extrapolation beyond centennial timescales is uncertain. In this study, we evaluate the consistency between three physicochemical characterization methods that are considered as proxies for biochar persistence—hydropyrolysis (HyPy), solid-state electric conductivity (SEC) and elemental analysis to obtain molar hydrogen:carbon ratios. We produced 42 biochars from straw and wood using a continuously operated pilot-scale auger reactor at temperatures ranging from 400 to 800°C under otherwise constant pyrolysis conditions. We then systematically analyzed the elemental composition, SEC and the fraction of biochar carbon that is resistant to HyPy (BCHyPy). Hydropyrolyis eliminates all free and covalently bound non-aromatic species and all aromatic species consisting of up to seven fused rings. Our results confirm that BCHyPy content increases with pyrolysis temperature and stabilizes above 600–680°C, reaching >90% of total carbon in high-temperature biochars. Similarly, SEC increased exponentially with pyrolysis severity, correlating strongly with BCHyPy and H/C molar ratio. The latter has so far been used to predict biochar persistence. Our findings from a controlled temperature series of biochars highlight that SEC and BCHyPy could be useful proxies for parameterizing multi-pool decay models of biochars produced in practice.
**Order of Authors:**	Nikolas Hagemann
	Hans-Peter Schmidt
	Thomas D. Bucheli
	Jannis Grafmueller
	Silvio Vosswinkel
	Volker Herdegen
	William Meredith
	Clement N. Uguna
	Colin E. Snape
**Additional Information:**	
**Question**	**Response**
**Financial Disclosure**<br><br>Enter a financial disclosure statement that describes the sources of funding for the	Yes

Powered by Editorial Manager® and ProduXion Manager® from Aries Systems Corporation

work included in this submission. Review the submission guidelines for detailed requirements. View published research articles from *PLOS ONE* for specific examples.<br><br>This statement is required for submission and **will appear in the published article** if the submission is accepted. Please make sure it is accurate.<br><br>**Funded studies**<br>Enter a statement with the following details:<br>• Initials of the authors who received each award<br>• Grant numbers awarded to each author<br>• The full name of each funder<br>• URL of each funder website<br>• Did the sponsors or funders play any role in the study design, data collection and analysis, decision to publish, or preparation of the manuscript?<br><br>Did you receive funding for this work?	
Please add funding details.<br>as follow-up to "**Financial Disclosure**<br><br>Enter a financial disclosure statement that describes the sources of funding for the work included in this submission. Review the submission guidelines for detailed requirements. View published research articles from *PLOS ONE* for specific examples.<br><br>This statement is required for submission and **will appear in the published article** if the submission is accepted. Please make sure it is accurate.	The research at the University of Nottingham was supported by: (i) the Biotechnology and Biological Sciences Research Council [BBSRC, the Biochar Demonstrator, grant number BB/V011596/1] as part of the UKRI Greenhouse Gas Removal programme and (ii) the Department of Energy Security and Net Zero (DESNZ) through the Direct Air Capture and Greenhouse Gas Removal Programme Phases 1 and 2 for the grant "Bio-waste to Biochar (B to B) via Hydrothermal Carbonisation and Post-Carbonisation". The research at Ithaka was funded by the Deutsche Forschungsgemeinschaft (DFG, German Research Foundation) – 467391808.

Powered by Editorial Manager® and ProduXion Manager® from Aries Systems Corporation

**Funded studies**<br>Enter a statement with the following details:<br>• Initials of the authors who received each award<br>• Grant numbers awarded to each author<br>• The full name of each funder<br>• URL of each funder website<br>• Did the sponsors or funders play any role in the study design, data collection and analysis, decision to publish, or preparation of the manuscript?<br><br>Did you receive funding for this work?"	
Please select the country of your main research funder (please select carefully as in some cases this is used in fee calculation).<br>as follow-up to "**Financial Disclosure**<br><br>Enter a financial disclosure statement that describes the sources of funding for the work included in this submission. Review the submission guidelines for detailed requirements. View published research articles from *PLOS ONE* for specific examples.<br><br>This statement is required for submission and **will appear in the published article** if the submission is accepted. Please make sure it is accurate.<br><br>**Funded studies**<br>Enter a statement with the following details:<br>• Initials of the authors who received each award<br>• Grant numbers awarded to each author<br>• The full name of each funder<br>• URL of each funder website<br>• Did the sponsors or funders play any role in the study design, data collection and analysis, decision to publish, or preparation of the manuscript?<br><br>Did you receive funding for this work?"	GERMANY - DE
**Competing Interests**	I have read the journal's policy and the authors of this manuscript have the following

Powered by Editorial Manager® and ProduXion Manager® from Aries Systems Corporation

Use the instructions below to enter a competing interest statement for this submission. On behalf of all authors, disclose any competing interests that could be perceived to bias this work—acknowledging all financial support and any other relevant financial or non-financial competing interests.<br><br>This statement is required for submission and **will appear in the published article** if the submission is accepted. Please make sure it is accurate and that any funding sources listed in your Funding Information later in the submission form are also declared in your Financial Disclosure statement.<br><br>View published research articles from *PLOS ONE* for specific examples.<br><br>**NO authors have competing interests**<br><br>Enter: *The authors have declared that no competing interests exist.*<br><br>**Authors with competing interests**<br><br>Enter competing interest details beginning with this statement:<br><br>*I have read the journal's policy and the authors of this manuscript have the following competing interests: [insert competing interests here]*<br><br>* typeset	competing interests: Hans-Peter Schmidt reports a relationship with Carbon Standards AG that includes: board membership. Nikolas Hagemann reports a relationship with Carbon Standards AG that includes: board<br>membership. All other authors have declared that no competing interests exist.
**Ethics Statement**<br><br>Enter an ethics statement for this submission. This statement is required if the study involved:<br><br>• Human participants<br>• Human specimens or tissue<br>• Vertebrate animals or cephalopods<br>• Vertebrate embryos or tissues	N/A

• Field research<br><br>Write "N/A" if the submission does not require an ethics statement.<br><br>General guidance is provided below. Consult the submission guidelines for detailed instructions. **Make sure that all information entered here is included in the Methods section of the manuscript.**	

Powered by Editorial Manager® and ProduXion Manager® from Aries Systems Corporation

**Format for specific study types**<br><br>**Human Subject Research (involving human participants and/or tissue)**<br>• Give the name of the institutional review board or ethics committee that approved the study<br>• Include the approval number and/or a statement indicating approval of this research<br>• Indicate the form of consent obtained (written/oral) or the reason that consent was not obtained (e.g. the data were analyzed anonymously)<br><br>**Animal Research (involving vertebrate animals, embryos or tissues)**<br>• Provide the name of the Institutional Animal Care and Use Committee (IACUC) or other relevant ethics board that reviewed the study protocol, and indicate whether they approved this research or granted a formal waiver of ethical approval<br>• Include an approval number if one was obtained<br>• If the study involved *non-human primates*, add *additional details* about animal welfare and steps taken to ameliorate suffering<br>• If anesthesia, euthanasia, or any kind of animal sacrifice is part of the study, include briefly which substances and/or methods were applied<br><br>**Field Research**<br><br>Include the following details if this study involves the collection of plant, animal, or other materials from a natural setting:<br>• Field permit number<br>• Name of the institution or relevant body that granted permission	
**Data Availability**<br><br>Authors are required to make all data underlying the findings described fully available, without restriction, and from the time of publication. PLOS allows rare exceptions to address legal and ethical concerns. See the PLOS Data Policy and FAQ for detailed information.	Yes - all data are fully available without restriction

Powered by Editorial Manager® and ProduXion Manager® from Aries Systems Corporation

A Data Availability Statement describing where the data can be found is required at submission. Your answers to this question constitute the Data Availability Statement and **will be published in the article**, if accepted.<br><br>**Important:** Stating 'data available on request from the author' is not sufficient. If your data are only available upon request, select 'No' for the first question and explain your exceptional situation in the text box.<br><br>Do the authors confirm that all data underlying the findings described in their manuscript are fully available without restriction?	
**Describe where the data may be found in full sentences. If you are copying our sample text, replace any instances of XXX with the appropriate details.**<br><br>• If the data are **held or will be held in a public repository**, include URLs, accession numbers or DOIs. If this information will only be available after acceptance, indicate this by ticking the box below. For example: *All XXX files are available from the XXX database (accession number(s) XXX, XXX.).*<br>• If the data are all contained **within the manuscript and/or Supporting Information files**, enter the following: *All relevant data are within the manuscript and its Supporting Information files.*<br>• If neither of these applies but you are able to provide **details of access elsewhere**, with or without limitations, please do so. For example:<br><br>*Data cannot be shared publicly because of [XXX]. Data are available from the XXX Institutional Data Access / Ethics Committee (contact via XXX) for researchers who meet the criteria for access to confidential data.*<br><br>*The data underlying the results presented in the study are available from (include the name of the third party*	All relevant data are within the manuscript and its Supporting Information files.

and contact information or URL).<br>• This text is appropriate if the data are owned by a third party and authors do not have permission to share the data.<br><br>* typeset	
Additional data availability information:	

Manuscript

Click here to access/download;Manuscript;Quantifying persistent aromatic carbon_v16_PlosOne.docx

Proxies for use in Biochar Decay Models: Hydropyrolysis, Electric Conductivity, and H/C_{org} molar ratio

Nikolas Hagemann[a,b*], Hans-Peter Schmidt[b], Thomas D. Bucheli[a], Jannis Grafmueller[b,c], Silvio Vosswinkel[d,e], Volker Herdegen[d], William Meredith[f], Clement N. Uguna[f] and Colin E. Snape[f]

[a]: Agroscope, Reckenholzstraße 191, 8046 Zurich, Switzerland, nikolas.hagemann@agroscope.admin.ch, thomas.bucheli@agroscope.admin.ch

[b]: Ithaka Institut, Ancienne Eglise 9, 1974 Arbaz, Switzerland, and Altmutterweg 21, 63773 Goldbach, Germany, hagemann@ithaka-institut.org, schmidt@ithaka-institut.org, grafmueller@ithaka-institut.org

[c]: Hochschule Offenburg, Badstraße 24, 77652 Offenburg, Germany, jannis.grafmueller@hs-offenburg.de

[d]: Technische Universität Bergakademie Freiberg, Freiberg, Germany, volker.herdegen@tun.tu-freiberg.de

[e]: Eurofins Umwelt-Ost GmbH, Lindenstraße 11, 09627 Bobritzsch-Hilbersdorf, Germany

[f]: University of Nottingham, Faculty of Engineering, University Park Nottingham, NG7 2TU, United Kingdom, William.Meredith@nottingham.ac.uk, Clement.Uguna@nottingham.ac.uk, Colin.Snape@nottingham.ac.uk

*corresponding author

For submission to PLOS One

Keywords: pyrogenic carbon, persistence, carbon dioxide removal, PyCCS,

Abstract

Biochar is a carbon-rich material produced via pyrolysis that is increasingly recognized for its role in carbon sequestration, particularly through its application in agriculture and materials. However, accurately predicting the long-term persistence of biochar in the environment remains challenging. While incubation trials have been widely used to assess biochar degradation, their extrapolation beyond centennial timescales is uncertain. In this study, we evaluate the consistency between three physicochemical characterization methods that are considered as proxies for biochar persistence—hydropyrolysis (HyPy), solid-state electric conductivity (SEC) and elemental analysis to obtain molar hydrogen:carbon ratios. We produced 42 biochars from straw and wood using a continuously operated pilot-scale auger reactor at temperatures ranging from 400 to 800°C under otherwise constant pyrolysis conditions. We then systematically analyzed the elemental composition, SEC and the fraction of biochar carbon that is resistant to HyPy (BC_{HyPy}). Hydropyrolyis eliminates all free and covalently bound non-aromatic species and all aromatic species consisting of up to seven fused rings. Our results confirm that BC_{HyPy} content increases with pyrolysis temperature and stabilizes above 600–680°C, reaching >90% of total carbon in high-temperature biochars. Similarly, SEC increased exponentially with pyrolysis severity, correlating strongly with BC_{HyPy} and H/C molar ratio. The latter has so far been used to predict biochar persistence. Our findings from a controlled temperature series of biochars highlight that SEC and BC_{HyPy} could be useful proxies for parameterizing multi-pool decay models of biochars produced in practice.

1. Introduction

1.1. Biochar persistence and incubation trials

Biochar is a pyrogenic carbonaceous material that is deliberately produced by biomass pyrolysis and used in a non-oxidative manner. To achieve carbon sequestration (pyrogenic carbon capture and storage, PyCCS) [1], biochar is applied as an additive in materials like concrete, asphalt, and composites or in agriculture, e.g., as a manure additive or as a nutrient carrier in slow-release fertilizers. These agricultural applications ultimately introduce biochar into soil, where the biochar-carbon is stored, which is a key aspect of PyCCS. Soil-applied biochar has been third-party certified as carbon dioxide removal (CDR) since 2020 [2-4]. To account for the climate effect of biochar-CDR, it is crucial to determine the quantity of biochar-carbon that stays sequestered at any point in time after its soil application. Therefore, it is necessary to predict the persistence of biochar in soil.

The incubation of biochar in soil or similar matrixes combined with quantifying CO_2 release is an intuitive and widespread approach to assessing biochar degradation. However, as shown by Azzi *et al.* (2024) and Sanei *et al.* (2025) [5, 6], biochar incubation studies that stretch at maximum over several years are not suitable to extrapolate biochar degradation beyond decades to centuries. Instead, multi-pool decay models based on the combination of data obtained from incubation studies, accelerated aging experiments, and physicochemical characterization are suggested, which includes spectroscopy, chromatography, microscopy and elemental analysis to calculate the hydrogen/carbon (H/C) molar ratio [6]. Here, we propose the quantification of biochar-carbon resisting hydropyrolysis (BC_{Hypy}) and the solid-state electric conductivity (SEC) of biochar as two novel physicochemical characterization methods to support the parametrization of such novel decay models for individual biochars.

Hydropyrolysis (HyPy) [7], a pyrolysis process conducted at 550 °C under high-pressure hydrogen (150 bar), is an analytical technique used to remove thermally labile carbon compounds. Gas chromatography-mass spectrometry (GC-MS) analysis of the volatilized

compounds suggests that HyPy predominantly releases species with fewer than eight condensed aromatic rings, though this observation may be constrained by the volatility and ionization characteristics required for GC-MS analysis [8]. The volatilized compounds included coronene, which is a molecule consisting of 7 fused benzene rings and 24 carbon atoms [9]. The carbon that is not volatilized under HyPy is referred to as BC_{HyPy} (i.e., black carbon after HyPy) or SPAC (stable polycyclic aromatic carbon), which is operationally defined as highly condensed carbon (>7 aromatic rings). This method was originally developed to remove sorbed organic carbon from historic charcoal samples and to thus avoid a "dilution" of the radiocarbon signature of the original pyrogenic carbon [10]. Process parameters were selected to avoid hydrogasification, i.e., the conversion of carbonaceous compounds into methane, and the formation of secondary char [7, 11].

Hydropyrolysis has been used to assess the thermal stability and composition of biochars since 2015 [12]. The thermally labile fraction of biochars, i.e., non-BC_{HyPy} including compounds with up to seven condensed aromatic rings, is considered to be more susceptible to (microbial) degradation than BC_{HyPy}, for which centennial persistence was postulated [13-15]. The BCHyPy content increases with pyrolysis severity and there is a strong inverse correlation between the increase in BC_{HyPy} content, with minimal change below 450°C, rapid growth between 500-700°C and a plateau at higher temperatures[12].

Solid-state electric conductivity arises from biochar's aromatic carbon structure, where conjugated π-electrons enable electron transfer [16]. Higher pyrolysis temperatures enhance this effect by increasing graphitic ordering and reducing resistivity [17]. To quantify SEC, a two-probe packed-bed technique is used, where electrical resistance is measured under applied compressive pressure, and conductivity is calculated based on resistivity and bed length [17-19]. While higher SEC has already been correlated with increased contaminant remediation [18], we here aim to test SEC as proxy for biochar persistence.

To test and eventually establish these two analytical parameters for biochar persistence evaluation, we systematically produced 42 biochars from straw and wood at increasing temperatures between 400 to 800 °C. We then tested the consistency between these two methods, but also with the H/C ratio, which has previously been used as a proxy to estimate biochar persistence due to its link to the pyrolysis temperature and hence to biochar persistence. Unlike previous studies, biochars were not produced in lab-based batch pyrolysis setups (e.g., thermo-gravimetric analysis, muffle furnace), but in a continuously operating auger pyrolysis reactor at a pilot scale of 1 kg biomass input per hour [20]. This setup provides conditions that are similar to most commercial pyrolysis units while allowing the application of a wide range of well-defined pyrolysis conditions such as temperature and residence time. We aimed for a correlation of BC_{HyPy}, SEC, pyrolysis temperature, and H/C molar ratio and to discuss the suitability of these parameters for the parametrization of novel decay models for industrial biochars dedicated to PyCCS.

2. Materials and Methods

2.1. Biochar production

Pellets were produced with a diameter of 6 mm on a roller wheel mill (WK230, EverTec, Groß-Zimmern, Germany) from straw (Jumbo/Coop, Basel, Switzerland, 5.9% ash content, cf. Tab. S1) and softwood (Allspan Spanverarbeitung GmbH, Karlsruhe, Germany, 0.4% ash content), respectively. Three batches each of straw and wood pellets were produced at different points in time from the same biomass (first batch: biochars produced at 400-600 °C, second batch: 620-800 °C, third batch: replicate biochars produced at 600 and 700 °C). Experimental pyrolysis was performed with a PYREKA research pyrolysis unit (Pyreg GmbH, Dörth, Germany), a continuously operated auger reactor [20] adjusted to a residence time of 10 min. Feeding rate was kept constant for each batch of feedstock and was in the range 0.4-0.7 kg h^{-1}. The reactor was purged with 2 L min^{-1} N_2. Biochars were collected for 30-45 min to achieve 50-150 g per

sample. After changing the pyrolysis temperature during continuous feeding of biomass into the reactor, biochar produced during the subsequent 30 min was discarded; when the input of a new biomass was started, the production of the first 45 min was discarded. We labeled the biochars beginning with the feedstock (i.e., W for wood, S for straw) followed by the pyrolysis temperature (e.g., a biochar produced at 600 °C from straw pellets is denoted as S600). If the temperature indication is followed by the capital letters A-C (e.g., W600A), it is a replicate of the biochar production (intra-day precision). The replicates were produced during an ongoing continuous pyrolysis, each with a sampling interval of 30 min.

2.2. Biochar characterization

Elemental analysis (CHN) was performed according to DIN 51732. The SEC was determined while the ground biochar (< 0.2 mm) was subjected to a pressure of 10 kN between two electrodes of the "Black Gauß I" device, which equals 30 MPa. The apparatus and procedure are described in detail elsewhere [19]. Both methods are compliant with the analytical guidelines of the European Biochar Certificate [21]. The ash content needed to express the BC_{HyPy} content on a dry and ash-free (daf) basis was quantified according to DIN DIN 51719 (550 °C). Oxygen (O) was determined on a vario EL-cube (elementar, Langenselbold, Germany).

For HyPy, 100-200 mg of biochar sample were loaded with a Mo catalyst using an aqueous/methanol 0.2 M solution of ammonium dioxydithiomolybdate [$(NH_4)_2MoO_2S_2$]. Catalyst weight was ~ 10% of the sample weight. The catalyst-loaded biochar samples were placed in quartz tubes (20 mm long), sealed with a sintered disc at the base, and placed in the HyPy reactor. The samples were heated at a rate of 300 °C min^{-1} from 50 to 250 °C, then heated at 8 °C min^{-1} from 250 °C until the final temperature of 550 °C, which was held then for 2 min under a hydrogen pressure of 15 MPa. More details are described elsewhere [11]. A controlled constant hydrogen sweep-gas flow of 5 L min^{-1} in the reactor, measured at ambient temperature

and pressure, ensured that the labile products were quickly removed from the samples. The mass and carbon content of the HyPy residue were quantified.

3. Results

Carbon contents of the woody biochars produced at 400-800 °C were between 81-92% and increased with increasing temperature (Table S2) while H/C molar ratios decreased from 0.43 to 0.10. Straw biochars had carbon contents of 62-70% with a peak at 550 °C, despite a continuously decreasing H/C molar ratio from 0.48 to 0.16 for 400-800 °C.

Solid-state electric conductivity increased exponentially with increasing pyrolysis temperature and ranged from 10^{-5} to 10^{3} mS cm^{-1} (Fig 1a). Notably, the straw biochars had consistently higher conductivity than woody biochars for the range of 400-700 °C, despite having lower carbon and lower BC_{HyPy} contents, indicating that other factors than pyrolysis temperature also affect conductivity.

Hydropyrolysis revealed a BC_{HyPy} fraction of 46.7% and 59.0% of total carbon (TC) for straw and wood biochar produced at 400°C, respectively. When pyrolyzed at 680 °C or above, BC_{HyPy} fraction of biochar from both feedstocks was >90%; for woody biochars, this value was already achieved at 600 °C (Table S2, Fig 1C). For temperatures above 600 °C, BC_{HyPy} content stabilized (varied) between 90-99% and 90-97% for woody and straw biochars, respectively (Table S2, Fig. 1c).

Both the SEC and the content of BC_{HyPy} increased with decreasing H/C molar ratio (Fig 1b, d). Whereas the content of BC_{HyPy} of straw and wood biochars was almost identical at a given H/C ratio, SEC was systematically higher for straw biochar, which consistently had higher molar H/C ratios for pyrolysis temperatures above 680 °C (Fig 1e). Woody biochar reached 90% BC_{HyPy} when SEC was above 0.1 mS cm^{-1}, and H/C below 0.27. Straw biochar reached 90% BC_{HyPy} only when SEC was above 26 mS cm^{-1} and the H/C below 0.20. Triplicate biochar production and analysis showed excellent repeatabilities for both feedstocks. Values of BC_{HyPy}

varied by approximately 1%, which is lower than the variation coefficient for H/C molar ratios, while the variation coefficient was 7-23% for SEC (Table 1).

Figure 1: Solid-state electric conductivity (SEC, mS cm^{-1}, a, b,) and BC_{HyPy} content in percent of total carbon (TC) of biochars (c, d, f) produced at defined temperatures in the range of 400-800 °C from straw and wood pellets. The pyrolysis temperature (a, c, e) and biochar H/C molar ratios (b, d) are used as parameters. Biochar H/C molar ratio is presented as a function of pyrolysis temperature (e).

Table 1: Properties of biochars produced in triplicates (A, B, C): content of total carbon (TC), hydrogen (H), H/C molar ratio, BC_{HyPy} as part of total carbon (TC). Biochars were produced from wood (W) and straw (S) pellets at 600 °C and 700 °C as indicated in the sample name. Variation coefficient (Var. Coeff.) is the quotient of the standard deviation and the mean value.

	C (%)	H (%)	H/C ratio		BC_{HyPy} (% of TC)		SEC (µS cm^{-1})	
				Var. Coeff		Var. Coeff		Var. Coeff
W600A	88.7	2.6	0.36		91.1		2.4 x 10^{-3}	
W600B	89.1	2.6	0.35	1.3%	91.5	1.0%	2.0 x 10^{-3}	7.3%
W600C	88.7	2.6	0.35		89.4		2.3 x 10^{-3}	
W700A	91.4	1.7	0.22		98.4		4.2 x 10^{1}	
W700B	91.2	1.8	0.23	2.1%	98.6	0.3%	3.6 x 10^{1}	7.5%
W700C	91.5	1.7	0.22		99.0		3.6 x 10^{1}	
S600A	68.7	2	0.35		88.5		7.6 x 10^{-2}	
S600B	70.5	2	0.34	1.4%	87.8	0.8%	1.1 x 10^{-1}	22.7%
S600C	70.8	2	0.34		86.7		7.0 x 10^{-2}	
S700A	71.2	1.4	0.24		95.5		1.2 x 10^{2}	
S700B	71.5	1.3	0.22	3.5%	96.2	0.8%	9.4 x 10^{1}	12.0%
S700C	71.4	1.4	0.23		94.4		1.3 x 10^{2}	

4. Discussion

4.1. Biochar properties determined by feedstock type, preparation, and pyrolysis conditions

Pyrolysis leads to the volatilization of low molecular weight carbonaceous compounds rich in O and H, resulting in an increase in carbon content and a decrease in H/C molar ratio of the solid product [12, 22], which was confirmed in the present study. However, when using straw, a high-ash biomass (5.9%, Tab. S1), the carbon content of the resulting biochars decreased at

temperatures above 550°C because of the non-proportional accumulation of mineral matter [12] which was not observed for the biochars made from low-ash wood (0.4%).

While low-temperature biochar is an electrical isolator, high-temperature biochar is electrically conductive due to the presence of conjugated π-electrons in its aromatic carbon structure. The degree of graphitization and aromaticity strongly influence conductivity, as a higher proportion of sp^2-hybridized carbon enhances charge transport. Biochars produced at higher pyrolysis temperatures exhibit increased conductivity due to larger graphitic ordering and π-electron delocalization as the result of a higher degree of polycondensation [23, 24]. X-ray diffraction and ^{13}C nuclear magnetic resonance spectroscopy revealed that aromaticity and the degree of polycondensation increase with pyrolysis temperature, which is further indicated by the reduction of the H/C molar ratio [22, 25-28]. The data on the SEC presented here fit well into the state of knowledge: at higher pyrolysis temperatures, biochar present a higher degree of polycondensation, which results in higher electrical conductivity. It should be emphasized that the increase occurred exponentially. In the parameter range investigated here (up to 800 °C and H/C = 0.1), a linear increase in the logarithmic SEC can be observed without any signs of saturation. For a better mechanistic understanding, future studies should include even higher pyrolysis temperatures and the measurement of reference materials such as conductive carbon black and defined nano carbon species.

The increase in the BC_{HyPy} content with increasing pyrolysis temperature and decreasing H/C molar ratios fits into the context of the literature presented above and confirms the previous HyPy studies with lab-produced biochars [7, 12, 13, 15]. Remarkably, the data show a saturation in BC_{HyPy} content when pyrolysis temperatures exceeded 600 °C and 680 °C for wood and straw, respectively, with BC_{HyPy} > 90% of TC. This is in line with the sigmoidal-like progression of BC_{HyPy} observed by McBeath and colleagues in pyrolysis experiments conducted at 300-900 °C [12]. Further experiments with higher pyrolysis temperatures and/or longer residence times should investigate in more detail if BC_{HyPy} plateaus > 90% TC or if

(virtually) all carbon in biochar can be BC_{HyPy}. The latter can be expected but is not observed so far, which may indicate an artefact or contamination in the analytical HyPy.

Howell and colleagues [15] suggested a limit of 75 wt% BC_{HyPy} in biochar, which was exceeded by several samples in this study, with up to 90.6 wt% BC_{HyPy}, representing as much as 99.0% of its total carbon content (W700C, Table 2). Interestingly, Howell and colleagues produced biochar from woody biomass at temperatures of up to 800°C and up to 10 min holding time (+ 100 °C /min heating rate), which at first glance would appear to be comparable to the conditions used in this study, as carbon speciation and aromaticity in particular is controlled by feedstock selection, (maximum) pyrolysis temperature and the residence time in the pyrolysis setup [26]. While the BC_{HyPy} content given as the percentage of the total biochar weight was lower in straw than in wood biochar, which is due to the higher ash content of the straw feedstock, feedstock selection had no consistent impact on the BC_{HyPy} when expressed as a ratio to the TC content of the biochar.

McBeath and colleagues quantified BC_{HyPy} in biochars produced at up to 900 °C from a broad range of biomasses, which covered ash content of 0.1-39.8% and suggested that higher ash content, and specifically the content of amorphous silica, may inhibit the formation of polycondensated structures and thus reduce BC_{HyPy}. They performed pyrolysis in batches of 20-200 g of biomass in a muffle furnace flushed with nitrogen and controlled the temperature in the biomass bed and held the desired temperature for 1 h. Their data on BC_{HyPy} of biochars from pine wood and corn stover is in good agreement to the data on biochar from softwood and straw presented in this study, respectively (Fig. 2a-b). For biochars produced at 500 °C or less, the present study showed higher BC_{HyPy}. In our study, the pyrolysis temperature was measured on the reactor wall. We observed selectively that the pyrolytic system no longer had to be actively heated when performing pyrolysis at 400-500 °C and that in some cases, temperatures above the set pyrolysis temperature were measured as the pyrolysis process was obviously exothermally in this temperature range. These observations were not systematically

documented but measured temperatures did not deviate more than 5-10% from the set pyrolysis temperature between 400-500 °C. Still, higher BC_{HyPy} content compared to McBeath *et al.* in the range of 400 to 500 °C might be the result of actually higher pyrolysis temperatures due to exothermal reactions.

Howell and colleagues used pulverized biomass sieved to < 0.425 mm, while considerably larger biomass pellets were used in the present study. They had a diameter of 6 mm and a length of approximately 5-10 mm (Fig S1). The biomass particle geometry affects the heating rate and gas exchange during pyrolysis and thus impacts biochar carbon speciation, which may explain the different results [29, 30]. Moreover, Howell et al. performed thermal treatment in thermogravimetric analyzer using only 100 mg biomass under inert gas or oxygen, which reduces secondary pyrolysis reactions [15]. The latter are known to result in highly aromatized carbon species, as demonstrated in industrial pyrolysis devices [31]. The resulting biochars consistently had lower BC_{HyPy} contents at similar H/C molar ratios compared to the biochars produced in our study (Fig. 2c). A high (>90 wt% daf) BCHyPy content was only achieved by Howell et al. when some type of gasification was performed (thermal treatment under the supply of oxygen that is not sufficient for full oxidation) [15, 32]. This highlights the need to conduct analyses of "real-life" industrial biochars. The use of a pilot plant in the present study was a compromise between practice-oriented, industrial-like pyrolysis conditions and the possibility of testing a range of pyrolysis temperatures under otherwise constant conditions.

Figure 2: Comparison of the content of BC_{HyPy} from biochar obtained from a continuously operating auger reactor (this study) with similar studies: McBeath et al. [12] pyrolyzed batches of 20-200g of pre-dried pine wood (a) and corn stover (b) under nitrogen flow in a muffle furnace. H/C molar ratio was not available in this study. Howell et al. (c) performed thermal treatment of pulverized woody biomass (<425 µm) in a thermogravimetric analyzer at 300-800 °C for 1-10 minutes under flow of air or nitrogen as an inert gas; only data points with H/C molar ratio < 0.7 were included.

4.2. Prospects and limits of using BC_{HyPy} in multi-pool decay models

It would be very appealing to determine the persistence of biochar in the environment via the pyrolysis temperature, as this would be (supposedly) simple and, above all, inexpensive. Our

data (Fig 2a) could be interpreted in this way, and the IPCC suggested a similar approach to estimate biochar persistence for national greenhouse gas inventories [33]. However, the reality is more complex: In our experiments on PYREKA, the temperature was the single difference in pyrolysis conditions, whereas other factors impacting biochar properties, such as reactor design, particle size of biomass, residence time of solid and gaseous pyrolysis products, and residual oxygen concentration in the reactor were constant [34]. As such, our findings to this end should not be considered universally applicable and cannot be directly extrapolated to comparisons of biochars derived from different feedstocks or subjected to varying production processes. In practice, different reactor designs and a wide range of pyrolysis conditions impact biochar properties despite the general consensus that pyrolysis temperature is the most important pyrolysis process determining biochar properties [15, 22, 26, 29, 32, 35]. Moreover, determining pyrolysis temperature in practice is often challenging to impossible due to moving parts in most reactors and the challenge of establishing ideal heat transfer between thermocouples and the biomass [15, 36]. Also, the heterogeneity of biochar (e.g., with regard to PAH contents) suggests considerable variability of temperature distribution within an industrial pyrolysis reactor [37]. Thus, pyrolysis temperature should not be used for the parametrization of decay models for individual biochars. Instead, biochar decay models must be parameterized by analytical data of the produced biochar. Robust and, in the best case, simple methods are needed to enable high-throughput analysis of biochar persistence.

Initially, HyPy was not designed to quantify a persistent biochar carbon fraction. Instead, its purpose was to isolate black carbon from organic impurities in environmental samples. The HyPy residue is an operationally defined, thermally stable carbon fraction (H/C < 0.5, >7-ring polycondensed clusters), which is quantified with high reproducibility and precision (triplicate measurements typically within ±2% variation coefficient). As there is strong consensus in the literature that highly condensed aromatic clusters in biochar can be considered persistent (but not inert) [38-40], BC_{HyPy} could be one factor to parameterize multi-pool decay models. Still,

HyPy does not measure the actual size and speciation of these aromatic clusters but provides an operationally defined threshold measure for the degree of poly-condensation. To study the speciation of the BC_{HyPy} fraction, X-ray diffraction [25, 26] can be employed and used for correlations with other biochar properties.

Electric conductivity could be an indicator for the speciation of BC_{HyPy}. SEC was in the range of 10^1-10^3 for biochars produced at 680-800 °C, which obviously differ in carbon speciation based on previous research [26], despite showing plateauing BC_{HyPy} at >90% TC. To better understand the role of SEC as an indicator of biochar persistence, further investigations are necessary to determine whether and to what extent SEC could also be influenced by parameters other than carbon speciation, e.g. the ash content. Solid state electric conductivity may be one of the easiest methods in biochar persistence analysis to perform with little experimental equipment required.

Non-BC_{HyPy} may include more or less alkanes, heterocyclic aromatic compounds, PAHs with up to seven aromatic rings, and their alkylated counterparts as revealed by GC-MS of the compounds volatilized during HyPy. It may also include some larger aromatic molecules that cannot be quantified with conventional GC/MS [8]. Its composition depends on feedstock, pyrolysis temperature, and further biochar production conditions [7, 9, 41]. Understanding the stability and fate of non-BC_{HyPy} in the environment is needed to quantify the time-dependent carbon sequestration of the less-persistent fraction of a given biochar [42]. It would allow to distinguish between the persistent, semi-persistent, and labile fractions of biochar and to derive a time-dependent carbon-sink accounting curve for the total biochar carbon applied to soil or materials.

Both SEC and BC_{HyPy} correlate well with the H/C molar ratio, which is currently used to approximate a stable carbon fraction in individual biochars based on incubation-derived data on biochar persistence [2-4]. Other studies correlate biochar persistence with its O/C molar ratio [15, 43]. However, the O content of biochar is usually calculated after quantifying C, H, N, S,

and ash content with insufficient precision [44], while the direct measurement is not standardized yet [21]. Thus, the determination of O content in praxis is less reliable than most other biochar properties.

Another approach to identify a persistent carbon fraction in biochar is the analysis of macerals (organic minerals) in the carbonaceous material according to guidelines of the International Committee for Coal and Organic Petrology – ICCP [45-48]. Here, light microscopy is used to identify structures in an embedded and polished biochar sample. The reflectance of visible light is then determined microscopically according to ISO 7404-5 (vitrinite reflectance) to quantify the content of the maceral inertinite, which is considered the most recalcitrant maceral. However, the name inertinite does not mean that this maceral is inert, but that it is far less reactive than others [49]. Future research should compare the different persistence proxies, including elemental analysis (H/C and O/C molar ratios), HyPy, SEC, vitrinite reflectance and other methods, preferably on industrial biochars. There is an urgent need to consolidate the findings from physico-chemical characterization of biochar [43, 50], from controlled incubation experiments [5, 40] and from field trials [51, 52] into a unified understanding of biochar persistence in the environment, as the current data and their interpretation are still perceived as contradictory.

5. Conclusion

Precise quantification of carbon sinks, both in terms of their size and their lifetime, is a prerequisite for a well-grounded deployment of negative emission technologies. This study suggests BC_{HyPy} and SEC as novel analytical tools to improve the assessment of biochar persistence. Our results indicate that both parameters correlate strongly with the pyrolysis temperature and the H/C molar ratio of experimental biochars produced under highly controlled conditions within a temperature gradient. This supports the use of these parameters as proxies for the persistence of biochars produced in practice. BC_{HyPy} quantification confirms that highly

condensed aromatic structures become dominant at pyrolysis temperatures above 600°C (for biochar from wood) and 680°C (for biochar from straw), in agreement with previous findings on biochar thermal stability. While pyrolysis temperature and feedstock selection remain key factors for biochar stability, our findings underscore the importance of additional process parameters, including reactor design and biomass particle size to be considered in future research. Simple biochar production parameters such as (highest treatment) temperature are not sufficient to predict biochar properties reliably.

Regarding HyPy and SEC, future studies should include pyrolysis temperatures above 800 °C and/or longer residence times to understand whether and when saturation occurs in the correlation of the H/C molar ratio, SEC, and HyPy. Solid-state electric conductivity and BC_{HyPy} should be quantified in biochars used in extended incubation studies and respective non-incubation retention samples. This would require characterization of the non-BC_{HyPy} fraction, e.g. by suitable GC-MS and/or ^{13}C nuclear magnetic resonance spectroscopy to quantify which special are actually degraded. The influence of biomass must also be investigated in more detail, especially that of biomass with higher ash contents. In the present study, straw was used, while potential feedstock such as digestate or sewage sludge has still higher ash contents.

The present study provides an impetus for the further development of multi-pool degradation models for biochar that will likely include further analytical methods. The presented results of HyPy and solid electric conductivity must now be compared and reconciled with other proposed characterization methods, in particular vitrinite reflectance.

Funding

The research at the University of Nottingham was supported by: (i) the Biotechnology and Biological Sciences Research Council [BBSRC, the Biochar Demonstrator, grant number BB/V011596/1] as part of the UKRI Greenhouse Gas Removal programme and (ii) the Department of Energy Security and Net Zero (DESNZ) through the Direct Air Capture and

Greenhouse Gas Removal Programme Phases 1 and 2 for the grant "Bio-waste to Biochar (B to B) via Hydrothermal Carbonisation and Post-Carbonisation". The research at Ithaka was funded by the Deutsche Forschungsgemeinschaft (DFG, German Research Foundation) – 467391808.

Author contributions: CRediT

NH: Conceptualization, Investigation, Writing - original draft

HPS: Investigation, Formal analysis, Writing - review & editing

TDB: Writing - review & editing

JG: Investigation, Writing - review & editing

SV: Investigation

VH: Writing - review & editing

WM: Investigation, Writing - review & editing

CU: Investigation, Writing - review & editing

CES: Funding acquisition, Conceptualization, Writing - review & editing

References:

[1.] Schmidt, H.-P., A. Anca-Couce, N. Hagemann, C. Werner, D. Gerten, W. Lucht, *et al.*, *Pyrogenic carbon capture and storage.* GCB Bioenergy, 2019. **11**(4): p. 573-591 http://doi.org/10.1111/gcbb.12553.

[2.] Etter, H., A. Vera, C. Aggarwal, M. Delaney, and S. Manley, *Methodology for biochar utilization in soil and non-soil applications. Available from: https://verra.org/wp-content/uploads/imported/methodologies/210803_VCS-Biochar-Methodology-v1.0-.pdf [last accessed: Mar 29, 2025].* 2021

[3.] EBC, *Certification of the carbon sink potential of biochar, Ithaka Institute, Arbaz, Switzerland. (http://european-biochar.org). Version 2.1E of 1st February 2021.* 2020

[4.] Puro.earth, *Puro standard- Biochar methodology, (Edition 2022 Version 2). Available from: https://fs.hubspotusercontent00.net/hubfs/7518557/Supplier%20Documents/Puro.earth%20Biochar%20Methodology.pdf. [last accessed: Mar 29, 2025].* 2022

[5.] Azzi, E.S., H. Li, H. Cederlund, E. Karltun, and C. Sundberg, *Modelling biochar long-term carbon storage in soil with harmonized analysis of decomposition data.* Geoderma, 2024. **441**: p. 116761 https://doi.org/10.1016/j.geoderma.2023.116761.

[6.] Sanei, H., H.I. Petersen, D. Chiaramonti, and O. Masek, *Evaluating the two-pool decay model for biochar carbon permanence.* Biochar, 2025. **7**(1): p. 9 https://doi.org/10.1007/s42773-024-00408-0.

[7.] Meredith, W., A.V. McBeath, P. Ascough, and M. Bird, *Analysis of biochars by hydropyrolysis*, in *Biochar: A Guide to Analytical Methods*, B. Singh, M. Camps-Arbestain, and J. Lehmann, Editors. 2017, Csiro Publishing. p. 320.

[8.] Poster, D.L., M.M. Schantz, L.C. Sander, and S.A. Wise, *Analysis of polycyclic aromatic hydrocarbons (PAHs) in environmental samples: a critical review of gas chromatographic (GC) methods.* Analytical and Bioanalytical Chemistry, 2006. **386**(4): p. 859-881 10.1007/s00216-006-0771-0.

[9.] Ascough, P.L., M.I. Bird, W. Meredith, R.E. Wood, C.E. Snape, F. Brock*, et al.*, *Hydropyrolysis: Implications for Radiocarbon Pretreatment and Characterization of Black Carbon.* Radiocarbon, 2010. **52**(3): p. 1336-1350 https://doi.org/10.1017/S0033822200046427.

[10.] Ascough, P.L., M.I. Bird, F. Brock, T.F.G. Higham, W. Meredith, C.E. Snape*, et al.*, *Hydropyrolysis as a new tool for radiocarbon pre-treatment and the quantification of black carbon.* Quaternary Geochronology, 2009. **4**(2): p. 140-147 https://doi.org/10.1016/j.quageo.2008.11.001.

[11.] Meredith, W., P.L. Ascough, M.I. Bird, D.J. Large, C.E. Snape, Y. Sun*, et al.*, *Assessment of hydropyrolysis as a method for the quantification of black carbon using standard reference materials.* Geochimica et Cosmochimica Acta, 2012. **97**: p. 131-147 https://doi.org/10.1016/j.gca.2012.08.037.

[12.] McBeath, A.V., C.M. Wurster, and M.I. Bird, *Influence of feedstock properties and pyrolysis conditions on biochar carbon stability as determined by hydrogen pyrolysis.* Biomass and Bioenergy, 2015. **73**: p. 155-173 https://doi.org/10.1016/j.biombioe.2014.12.022.

[13.] Rombolà, A.G., D. Fabbri, W. Meredith, C.E. Snape, and A. Dieguez-Alonso, *Molecular characterization of the thermally labile fraction of biochar by hydropyrolysis and pyrolysis-GC/MS.* Journal of Analytical and Applied Pyrolysis, 2016. **121**: p. 230-239 https://doi.org/10.1016/j.jaap.2016.08.003.

[14.] Cerniglia, C.E., *Biodegradation of polycyclic aromatic hydrocarbons.* Biodegradation, 1992. **3**(2): p. 351-368 https://doi.org/10.1007/BF00129093.

[15.] Howell, A., S. Helmkamp, and E. Belmont, *Stable polycyclic aromatic carbon (SPAC) formation in wildfire chars and engineered biochars.* Science of The Total Environment, 2022. **849**: p. 157610 https://doi.org/10.1016/j.scitotenv.2022.157610.

[16.] Sun, T., B.D. Levin, J.J. Guzman, A. Enders, D.A. Muller, L.T. Angenent*, et al.*, *Rapid electron transfer by the carbon matrix in natural pyrogenic carbon.* Nat Commun, 2017. **8**: p. 14873 https://doi.org/10.1038/ncomms14873.

[17.] Mochidzuki, K., F. Soutric, K. Tadokoro, M.J.J. Antal, M. Tóth, B. Zelei*, et al.*, *Electrical and Physical Properties of Carbonized Charcoals.* Industrial & Engineering Chemistry Research, 2003. **42**(21): p. 5140-5151

[18.] Xu, W., J.J. Pignatello, and W.A. Mitch, *Role of Black Carbon Electrical Conductivity in Mediating Hexahydro-1,3,5-trinitro-1,3,5-triazine (RDX) Transformation on Carbon Surfaces by Sulfides.* Environmental Science & Technology, 2013. **47**(13): p. 7129-7136 https://doi.org/10.1021/es4012367.

[19.] Eurofins-Umwelt-Ost-GmbH, *Conductivity meter Black Gauss I - technical documentation.* 2023 https://doi.org/10.5281/zenodo.8197758.

[20.] Hagemann, N., H.P. Schmidt, R. Kagi, M. Bohler, G. Sigmund, A. Maccagnan*, et al.*, *Wood-based activated biochar to eliminate organic micropollutants from biologically treated wastewater.* Sci Total Environ, 2020. **730**: p. 138417 http://doi.org/10.1016/j.scitotenv.2020.138417.

[21.] EBC, *European Biochar Certificate - Guidelines for a sustainable production of biochar. European Biochar Foundation (EBC), Arbaz, Switzerland.* www.european-biochar.org*. Version 10.3E of 5th Apr 2023.* 2012-2024

[22.] Ippolito, J.A., L. Cui, C. Kammann, N. Wrage-Mönnig, J.M. Estavillo, T. Fuertes-Mendizabal*, et al.*, *Feedstock choice, pyrolysis temperature and type influence biochar characteristics: a comprehensive meta-data analysis review.* Biochar, 2020 https://doi.org/10.1007/s42773-020-00067-x.

[23.] Kelemen, S.R., K.D. Rose, and P.J. Kwiatek, *Carbon aromaticity based on XPS II to II* signal intensity.* Applied Surface Science, 1993. **64**(2): p. 167-174 https://doi.org/10.1016/0169-4332(93)90276-H.

[24.] Pantea, D., H. Darmstadt, S. Kaliaguine, and C. Roy, *Electrical conductivity of conductive carbon blacks: influence of surface chemistry and topology.* Applied Surface Science, 2003. **217**(1): p. 181-193 https://doi.org/10.1016/S0169-4332(03)00550-6.

[25.] Keiluweit, M., P.S. Nico, M.G. Johnson, and M. Kleber, *Dynamic molecular structure of plant biomass-derived black carbon (biochar).* Environmental Science & Technology, 2010. **44**: p. 1247-1253 http://doi.org/10.1021/es9031419.

[26.] Hockaday, W.C., M. Kleber, and P.S. Nico, *Characteristics of biochar: Macromolecular properties*, in *Biochar for Environmental Management*, J. Lehmann and S. Joseph, Editors. 2024, Routledge: London and New York. p. 153-181.

[27.] Xiao, X., Z. Chen, and B. Chen, *H/C atomic ratio as a smart linkage between pyrolytic temperatures, aromatic clusters and sorption properties of biochars derived from diverse precursory materials.* Scientific Reports, 2016. **6**(1): p. 22644 https://doi.org/10.1038/srep22644.

[28.] Cao, X., J.J. Pignatello, Y. Li, C. Lattao, M.A. Chappell, N. Chen*, et al.*, *Characterization of Wood Chars Produced at Different Temperatures Using Advanced Solid-State 13C NMR Spectroscopic Techniques.* Energy & Fuels, 2012. **26**(9): p. 5983-5991 https://doi.org/10.1021/ef300947s.

[29.] Di Blasi, C., C. Branca, V. Lombardi, P. Ciappa, and C. Di Giacomo, *Effects of Particle Size and Density on the Packed-Bed Pyrolysis of Wood.* Energy & Fuels, 2013. **27**(11): p. 6781-6791 https://doi.org/10.1021/ef401481j.

[30.] Kaltschmitt, M., H. Hartmann, and H. Hofbauer, *Energie aus Biomasse - Grundlagen, Techniken und Verfahren* 2ed. 2009, Berlin, Heidelbaerg: Springer.

[31.] Anca-Couce, A., A. Dieguez-Alonso, N. Zobel, A. Berger, N. Kienzl, and F. Behrendt, *Influence of heterogeneous secondary reactions during slow pyrolysis on char oxidation reactivity of woody biomass.* Energy & Fuels, 2017. **31**(3): p. 2335-2344 http://doi.org/10.1021/acs.energyfuels.6b02350.

[32.] Hagemann, N., K. Spokas, H.-P. Schmidt, R. Kägi, M.A. Böhler, and T.D. Bucheli, *Activated carbon, biochar and charcoal: linkages and synergies across pyrogenic carbon's ABCs.* Water, 2018. **10**(2): p. 182 https://doi.org/10.3390/w10020182.

[33.] IPCC, *2019 Refinement to the 2006 IPCC Guidelines for National Greenhouse Gas Inventories - Appendix 4: Method for Estimating the Change in Mineral Soil Organic Carbon Stocks from Biochar Amendments: Basis for Future Methodological Development*. 2019.

[34.] Lebrun Thauront, J., G. Soja, H.-P. Schmidt, and S. Abiven, *A critical re-analysis of biochar properties prediction from production parameters and elemental analysis.* GCB Bioenergy, 2024. **16**(11): p. e13170 https://doi.org/10.1111/gcbb.13170.

[35.] Santin, C., S.H. Doerr, A. Merino, T.D. Bucheli, R. Bryant, P. Ascough*, et al.*, *Carbon sequestration potential and physicochemical properties differ between wildfire charcoals and slow-pyrolysis biochars.* Sci Rep, 2017. **7**(1): p. 11233 https://doi.org/10.1038/s41598-017-10455-2.

[36.] Shannon, K. and B. Butler. *A review of error associated with thermocouple temperature measurement in fire environments*. in *Proceedings of the 2nd Fire Ecology Congress*. 2003.

[37.] Bucheli, T.D., H.J. Bachmann, F. Blum, D. Buerge, R. Giger, I. Hilber*, et al.*, *On the heterogeneity of biochar and consequences for its representative sampling.* Journal of Analytical and Applied Pyrolysis, 2014. **107**: p. 25-30 https://doi.org/10.1016/j.jaap.2014.01.020.

[38.] Cheng, C.-H., J. Lehmann, J.E. Thies, S.D. Burton, and M.H. Engelhard, *Oxidation of black carbon by biotic and abiotic processes.* Organic Geochemistry, 2006. **37**(11): p. 1477-1488 https://doi.org/10.1016/j.orggeochem.2006.06.022.

[39.] Ascough, P.L., F. Brock, M.E. Collinson, J.D. Painter, D.W. Lane, and M.I. Bird, *Chemical Characteristics of Macroscopic Pyrogenic Carbon Following Millennial-Scale Environmental Exposure.* Frontiers in Environmental Science, 2020. **7** https://doi.org/10.3389/fenvs.2019.00203.

[40.] Zimmerman, A.R., *Abiotic and Microbial Oxidation of Laboratory-Produced Black Carbon (Biochar).* Environmental Science & Technology, 2010. **44**(4): p. 1295-1301 https://doi.org/10.1021/es903140c.

[41.] Rombolà, A.G., W. Meredith, C.E. Snape, S. Baronti, L. Genesio, F.P. Vaccari*, et al.*, *Fate of Soil Organic Carbon and Polycyclic Aromatic Hydrocarbons in a Vineyard Soil Treated with Biochar.* Environmental Science & Technology, 2015. **49**(18): p. 11037-11044 https://doi.org/10.1021/acs.est.5b02562.

[42.] Schmidt, H.-P., S. Abiven, N. Hagemann, and J. Meyer zu Drewer, *Permanence of soil applied biochar.* the Biochar Journal, 2022. **www.biochar-journal.org/en/ct/109,** : p. 69-74

[43.] Spokas, K.A., *Review of the stability of biochar in soils: predictability of O:C molar ratios.* Carbon Management, 2010. **1**(2): p. 289-303 https://doi.org/10.4155/cmt.10.32.

[44.] Bachmann, H.J., T.D. Bucheli, A. Dieguez-Alonso, D. Fabbri, H. Knicker, H.-P. Schmidt*, et al.*, *Toward the Standardization of Biochar Analysis: The COST Action TD1107 Interlaboratory Comparison.* Journal of Agricultural and Food Chemistry, 2016. **64**(2): p. 513-527 https://doi.org/10.1021/acs.jafc.5b05055.

[45.] Sanei, H., A. Rudra, Z.M.M. Przyswitt, S. Kousted, M.B. Sindlev, X. Zheng*, et al.*, *Assessing biochar's permanence: An inertinite benchmark.* International Journal of Coal Geology, 2024. **281**: p. 104409 https://doi.org/10.1016/j.coal.2023.104409.

[46.] Jones, T.P., A.C. Scott, and M. Cope, *Reflectance measurements and the temperature of formation of modern charcoals and implications for studies of fusain.* Bulletin de la Société Géologique de France, 1991. **162**(2): p. 193-200

[47.] Petersen, H.I., L. Lassen, A. Rudra, L.X. Nguyen, P.T.M. Do, and H. Sanei, *Carbon stability and morphotype composition of biochars from feedstocks in the Mekong Delta, Vietnam.* International Journal of Coal Geology, 2023. **271**: p. 104233 https://doi.org/10.1016/j.coal.2023.104233.

[48.] *The new inertinite classification (ICCP System 1994).* Fuel, 2001. **80**(4): p. 459-471 https://doi.org/10.1016/S0016-2361(00)00102-2.

[49.] ICCP, *International Committee for Coal Petrology: International Handbook of Coal Petrology*. 2nd ed. 1963, Paris: CENTRE NATIONAL DE AL RECHERCHE SCIENTIFIQUE.

[50.] Rudra, A., H.I. Petersen, and H. Sanei, *Molecular characterization of biochar and the relation to carbon permanence.* International Journal of Coal Geology, 2024. **291**: p. 104565 https://doi.org/10.1016/j.coal.2024.104565.

[51.] Lyu, J. and A.R. Zimmerman, *Large Losses of Pyrogenic Carbon (Biochar) and Native Soil Carbon During a 15-Month Field Study in North Florida, USA.* Agriculture, 2025. **15**(3): p. 300 https://doi.org/10.3390/agriculture15030300.

[52.] Gross, A., T. Bromm, S. Polifka, D. Fischer, and B. Glaser, *Long-term biochar and soil organic carbon stability – Evidence from field experiments in Germany.* Science of The Total Environment, 2024. **954**: p. 176340 https://doi.org/10.1016/j.scitotenv.2024.176340.

Figure 1

Click here to access/download;Figure;Hagemann_etal_Fig1.tiff

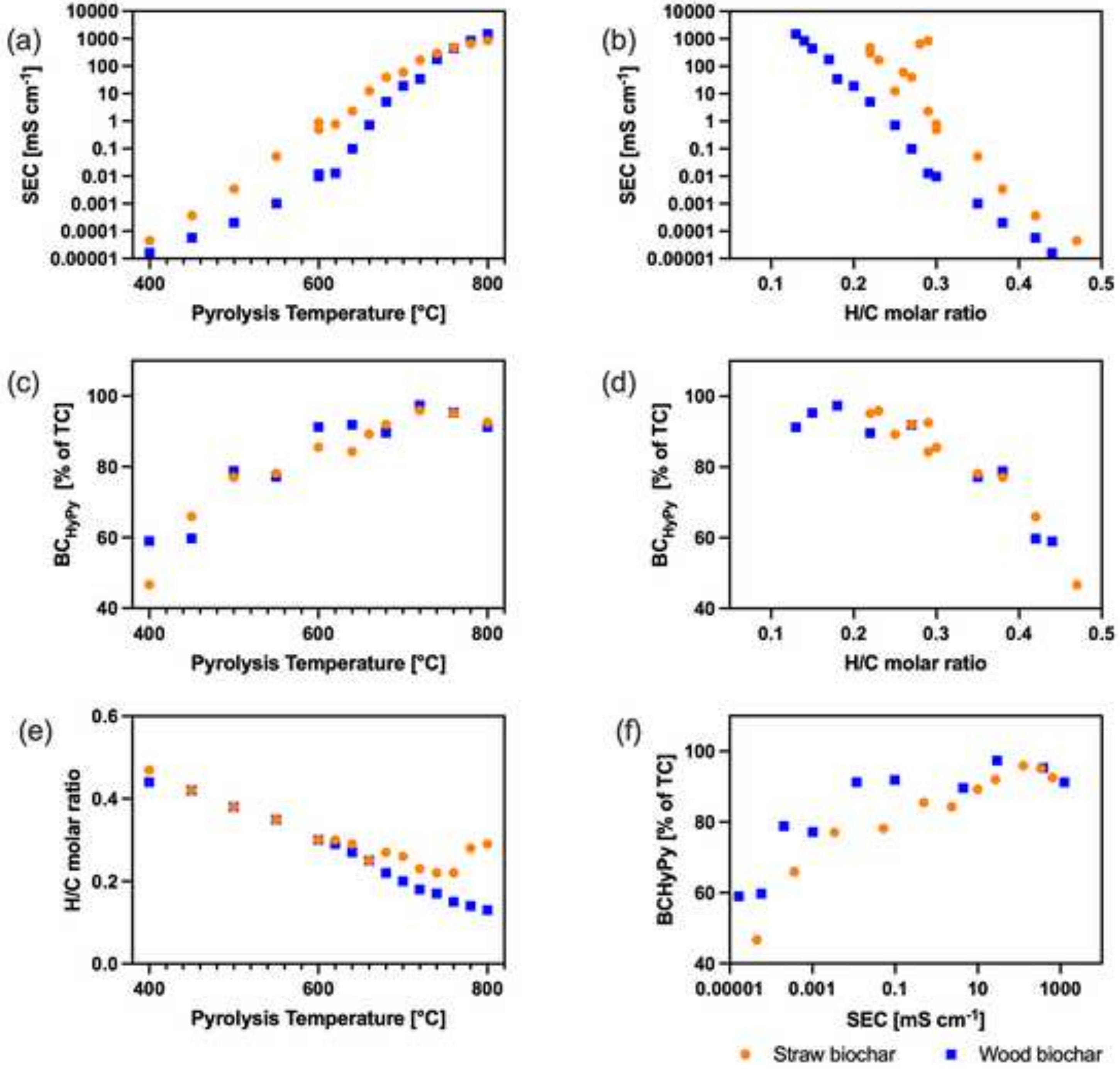

Figure 2 Click here to access/download;Figure;Hagemann_etal_Fig2.tiff

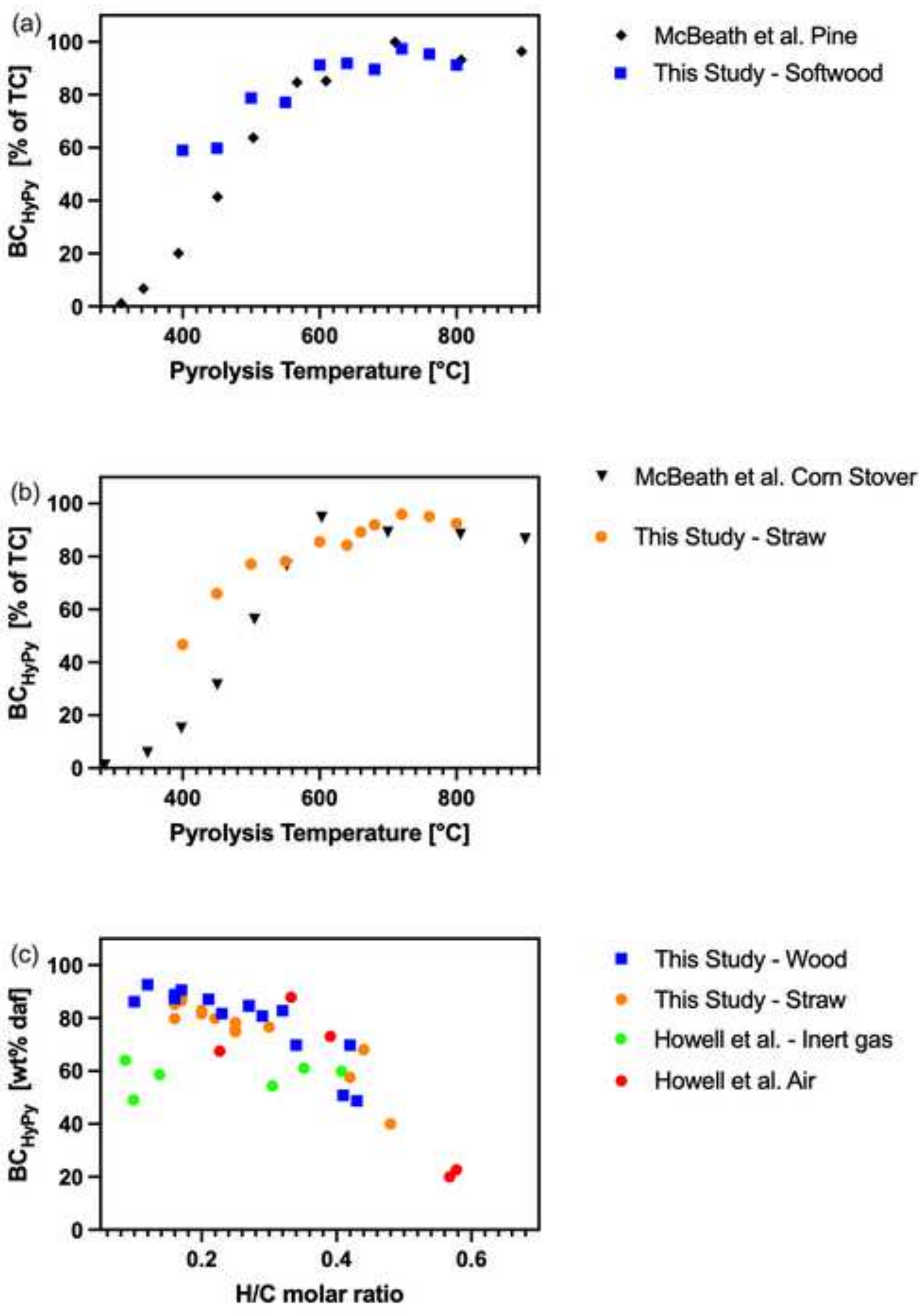

Click here to access/download
Supporting Information
Hagemann_etal_SI.pdf
